# 水通道蛋白3在非小细胞肺癌中的表达及与临床病理相关性的研究

**DOI:** 10.3779/j.issn.1009-3419.2012.07.03

**Published:** 2012-07-20

**Authors:** 白翎 李, 磊 金, 铿 钟, 大海 杜

**Affiliations:** 1 200433 上海，第二军医大学附属长海医院胸心外科 Department of Cardiothoracic Surgery, Changhai Hospital, the Second Military Medical University, Shanghai 200433, China; 2 200433 上海，第二军医大学附属长海医院实验诊断科 Department of Clinical Laboratory, Changhai Hospital, the Second Military Medical University, Shanghai 200433, China

**Keywords:** 肺肿瘤, AQP3, 微血管密度, 免疫组织化学, Lung neoplasms, AQP3, Microvessel density, Immunohistochemistry

## Abstract

**背景与目的:**

肺癌是严重威胁人类生存和发展的恶性疾病之一，本研究旨在探讨非小细胞肺癌（non-small cell lung cancer, NSCLC）组织中水通道蛋白-3（aquaporins 3, AQP3）的表达，探讨其与NSCLC临床病理学之间的关系。

**方法:**

应用免疫组织化学方法检测180例NSCLC组织中AQP3表达及微血管密度（micro vascular density, MVD）。

**结果:**

NSCLC组织中AQP3阴性表达率为13.9%（25/180），中等强度阳性表达率为37.2%（67/180），强阳性表达率为48.9%（88/180）。NSCLC组织中AQP3表达高于癌旁组织，有明显统计学差异（*P* < 0.01）。AQP3高表达也同时伴随着MVD计数增高（*P* < 0.01）。男性患者AQP3表达高于女性患者（*P*=0.003）。腺癌中AQP3的表达较鳞癌明显增强（*P* < 0.001）；有淋巴结转移的病例存在AQP3高表达（*P*=0.026）。NSCLC中AQP3的阳性表达率与肿瘤分化程度呈正相关，表现为AQP3的阳性表达率在高分化癌中明显高于低分化癌（*P* < 0.001）。

**结论:**

AQP3在NSCLC的肿瘤血管生成和进展中起重要促进作用，AQP3可能为NSCLC治疗的新靶点。

## Abstract (en)

**Background and objective:**

Lung cancer is a major health problem worldwide. The aim of this study is to investigate aquaporin 3 (AQP3) expression and its relationship with the clinicopathologic characteristics of non-small cell lung cancer (NSCLC).

**Methods:**

AQP3 expression and the microvascular density (MVD) of tissue samples from 180 cases with NSCLC were detected by immunohistochemistry.

**Results:**

AQP3 expression was negative in 25 (13.9%), positive in 67 (37.2%), and strongly positive in 88 (48.9%) of the 180 cases, which was significantly higher than that in the normal tissue (*P* < 0.01). A significant correlation was found between AQP3 expression and MVD (*P* < 0.01), whereas a high MVD was found among patients with strongly positive AQP3 expression. Male patients with positive or strongly positive AQP3 expression had significantly higher expression than female patients did (*P*=0.003). AQP3 expression was more significantly enhanced in adenocarcinoma than that in squamous cell carcinoma (*P* < 0.001). Statistical analysis indicated that the positive rate of AQP3 expression in well-differentiated carcinoma was significantly higher than that in poorly differentiated tumors (*P* < 0.001). Lymph node metastasis was positively correlated with high AQP3 expression (*P*=0.026).

**Conclusion:**

AQP3 expression was closely correlated with MVD in NSCLC, whereas high MVD was frequently found in tumors with high AQP3 expression. AQP3, as a therapeutic target for inhibiting high AQP3 expression in NSCLC tissues, may weaken cancer cell proliferation, invasion, and metastasis.

水通道蛋白（aquaporins, AQPs）是一组分子量约30 kDa的（单体）疏水性膜转运蛋白，属于主要固有蛋白（major intrinsic protein, MIP）家族^[1]^。近年来，AQPs的表型及临床病理相关研究取得了重大进展^[2]^。其中AQPs的染色体定位、基因结构、表达调控、蛋白构象、组织分布和生理功能方面研究较为深入，但AQPs功能对肿瘤的影响至今尚未明确。最近研究^[3]^发现AQP3在肿瘤细胞的增殖、侵袭、转移和血管生成中发挥了重要的作用。已有研究^[4]^证实AQP3在多种不同来源的肿瘤中表达水平较高，尤其是在侵袭性强的肿瘤中。但AQP3在非小细胞肺癌（non-small cell lung cancer, NSCLC）组织中的表达及与临床病理参数间的关系，国内外鲜有报道。本研究应用免疫组化法检测NSCLC中AQP3的表达情况，并分析其与微血管密度（micro vascular density, MVD）及临床病理参数间的相关性，为进一步研究AQP3在NSCLC发生、发展中的作用机制提供依据。

## 1 对象与方法

### 1.1 对象

选取2006年1月-2006年12月上海长海医院胸心外科手术切除的NSCLC标本180例，术前未行任何抗肿瘤治疗；其中男性117例，女性63例。患者年龄43岁-79岁，平均年龄54.7岁。病理分型为鳞癌64例，腺癌99例，大细胞癌10例，其它病理类型7例。所有标本均经过病理组织学诊断证实，癌旁组织作为对照组。

### 1.2 方法

#### 1.2.1 主要试剂

AQP3免疫组化试剂盒购自美国Santa Cruz公司；即用型SABC试剂盒及DAB显色剂购自武汉博士德公司，CD34单克隆抗体购自美国Santa Cruz公司。

#### 1.2.2 免疫组织化学分析

免疫组化染色采用SABC（streptavidin-biotin complex）法，高温抗原修复，染色程序参照说明书进行。用已知阳性切片作为阳性对照，用PBS缓冲液代替一抗作阴性对照。

### 1.3 结果判定

采用双盲法，由两位病理科医师分别读片。AQP3阳性判断：在胞膜、胞浆呈现棕黄色或棕褐色。每张切片随机选择5个不同视野计数，结果取其平均值。AQP3免疫组化评分方法：选择5个高倍视野（×200）计数细胞总数和阳性细胞数，得出阳性细胞百分率，其中：阳性细胞数 < 5%为阴性，计0分；5%-25%为弱阳性，计1分；26%-50%为中等强度阳性，计2分； > 50%为强阳性，计3分。按染色强度：弱为0分，中为1分，强为2分，共3级。总评分值=阳性细胞比例评分+染色强度评分；总评分值≤2分为阴性，3分-4分为中等强度阳性（+），5分-6分为强阳性（++）。

### 1.4 MVD计数

采用CD34染色计数MVD值。CD34定位于血管内皮细胞膜和胞浆，阳性者呈棕色。肿瘤MVD计数按照Weidner^[5]^方法进行。

### 1.5 统计学分析

所有数据均采用SPSS 11.0统计软件进行分析。采用方差分析（方差齐*LSD-t*检验，方差不齐*Tamhane*检验）比较不同组织中AQP3蛋白阳性染色结果总评分，率的比较采用χ^2^检验。以*P* < 0.05为差异有统计学意义。

## 2 结果

### 2.1 NSCLC组织、癌旁组织中AQP3的表达

AQP蛋白阳性表达主要定位于胞膜、胞浆，呈现棕黄色或棕褐色。正常肺组织中，AQP3分布于肺泡Ⅱ型上皮细胞、气道粘液上皮细胞胞质和顶膜及部分粘液腺细胞基底。免疫组化结果显示：NSCLC组织中AQP3阴性表达率为13.9%（25/180），中等强度阳性表达率为37.2%（67/180），强阳性表达率为48.9%（88/180）。NSCLC组织中AQP3表达高于癌旁组织（图 1），有明显统计学差异（*P* < 0.01）。

**1:**
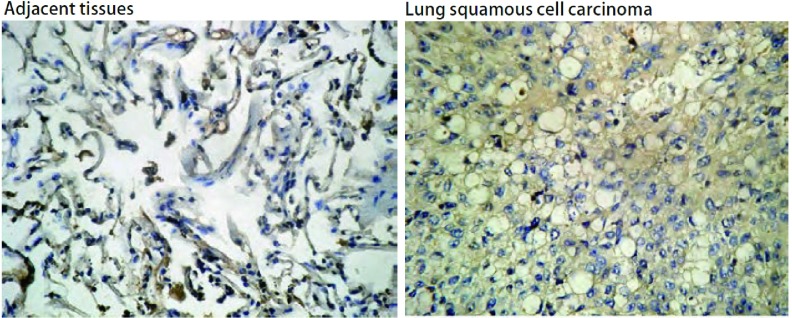
AQP3在NSCLC及癌旁组织中的表达（SABC, ×200） Expression of AQP3 in NSCLC and adjacent tissues (SABC, ×200). NSCLC: non-small cell lung cancer.

### 2.2 MVD与AQP3表达的相关性研究

CD34染色定位于血管内皮细胞，呈棕黄色。肿瘤间质中见大量血管内皮细胞阳性染色，微血管分布不均（图 2）。NSCLC组织中MVD计数为51.2±21.6，明显高于癌旁组织15.2±3.1（*P* < 0.01）。根据AQP3在NSCLC中的不同表达水平，分析其对MVD计数的影响，研究其相关性（表 1）。结果显示AQP3的表达与MVD表达密切相关，AQP3高表达的同时伴随着MVD计数增高（*P* < 0.01）。

**2:**
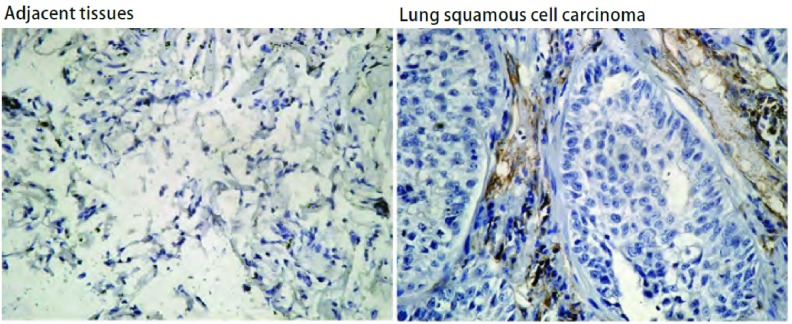
肺癌及癌旁组织中的MVD计数（SABC, ×200） The MVD counts in NSCLC and adjacent tissues (SABC, ×200)

**1:** MVD与AQP3表达的相关性分析 MVD according to the combination of AQP3 expressions

AQP3	*n*	MVD	*P*
-	25	33.5±6.9	*P* < 0.01
+	67	45.2±13.4	
++	88	62.7±21.6	
MVD: micro vascular density; AQP3: aquaporin 3.			

### 2.3 NSCLC组织中AQP3的表达与临床病理参数的关系

表 2显示了AQP3表达和NSCLC临床病理参数之间的关系。男性患者AQP3中等强度表达37例（31.6%），强阳性表达65例（55.6%），明显高于女性患者（*P*=0.003）。与鳞癌相比，腺癌中AQP3表达水平明显较高，其中中等强度表达29例（29.2%），强阳性表达65例（65.7%）（*P* < 0.001）；并且，发生淋巴结转移的病例往往伴随着AQP3的高表达（*P*=0.026）。NSCLC中AQP3的表达与肿瘤分化程度呈明显正相关，表现为AQP3的阳性表达率在高分化癌中明显高于低分化癌（*P* < 0.001）。但AQP3的表达情况与年龄、TNM分级等无相关性（*P* > 0.05）。

**2:** NSCLC中AQP3的表达及与临床病理特征的关系 AQP3 expression and clinicopathological factors in NSCLC

	*n*	AQP3			*χ*^2^	*P*
		(-) (*n*=25)	(+) (*n*=67)	(++) (*n*=88)		
Age (yr)					0.558	0.756
< 60	82	13 (15.8%)	29 (35.4%)	40 (48.8%)		
≥60	98	12 (12.2%)	38 (38.8%)	48 (49.0%)		
Gender					11.595	0.003
Male	117	15 (12.8%)	37 (31.6%)	65 (55.6%)		
Female	63	10 (15.9%)	30 (47.6%)	23 (36.5%)		
Histological type					67.836	< 0.001
Squamous cell carcinoma	64	6 (9.3%)	35 (54.7%)	23 (36.0%)		
Adenocarcinoma	99	5 (5.0%)	29 (29.2%)	65 (65.7%)		
Large cell	10	8 (80.0%)	2 (20.0%)	0		
Others	7	6 (85.7%)	1 (14.3%)	0		
Lymph node metastasis					7.301	0.026
Absent	53	8 (15.1%)	27 (51.0%)	18 (33.9%)		
Present	127	17 (13.4%)	40 (31.5%)	70 (55.1%)		
Tumor cell differentiation					33.559	< 0.001
Well-moderately differentiated	109	8 (7.3%)	29 (26.6%)	72 (66.1%)		
Poorly differentiated	71	17 (23.9%)	38 (53.5%)	16 (22.6%)		
Pathological stage (TNM)					0.409	0.815
Ⅰ/Ⅱ	104	13 (10.0%)	39 (35.0%)	52 (55.0%)		
Ⅲ	76	12 (15.8%)	28 (36.8%)	36 (47.4%)		

## 3 讨论

AQP是一种内在膜蛋白，AQP3是AQP家族成员之一，是第1个被发现的除了对水有通透性以外，还能够通透甘油和尿素的AQP。最新研究^[6]^发现AQP3参与了肿瘤的血管生成过程。人们已经发现起源于脑、胃、肾、结直肠等至少12种不同来源的人类肿瘤细胞有AQP3表达^[1]^，本研究结果显示人类NSCLC细胞亦存在AQPs家族中的AQP3高表达，临床病理分析表明AQP3可能参与NSCLC的血管生成。

恶性肿瘤基本的生物学特征为肿瘤细胞的无限增殖和分化异常。为满足快速增殖、分裂和侵袭转移的需要，肿瘤细胞比正常细胞更需要水分子的快速跨膜转运。多数恶性肿瘤有很高的组织间隙液体压力和微血管渗透性，细胞内外渗透压变化频率加快，这在肿瘤的侵袭和转移中具有重要作用^[7]^。AQP3表达或功能的改变不仅对水分子的运输产生明显影响，而且对细胞的生命活动至关重要。有研究^[8]^表明敲除*AQP3*基因，小鼠内皮细胞迁移速度明显降低，从而引起肿瘤血管生成障碍，肿瘤生长受抑制，提示AQP3与肿瘤血管生成及肿瘤生长、扩散有关。AQP3可以通过两种途径间接刺激肿瘤血管增生：①它与血管内皮生长因子（vascular endothelial growth factor, VEGF）起协同作用，使肿瘤血管具有很高的通透性，并且加强纤维素外渗，加强了内皮细胞的增殖和迁移，而这两种细胞功能促进了肿瘤血管生成和发展；②AQP3介导的高渗透性引起流体静力压的增高，并且随之引起缺氧，刺激血管生成^[9]^。

本研究对180例NSCLC标本进行了免疫组织化学分析；其中鳞癌64例，腺癌99例，大细胞癌10例，其它病理类型7例。研究发现，腺癌中AQP3强阳性率最高（65.7%），而鳞癌为35.9%，大细胞癌未检测到AQP3强阳性表达。因此表明，AQP3在肺组织的表达不仅具有细胞特异性，而且还具有肿瘤组织类型特异性。另外，对NSCLC肿瘤分化程度与AQP3表达关系的研究表明，高分化癌组织AQP3表达较多，低分化癌表达少。由于不同类型肺癌在肿瘤演进过程中可能存在着不同机制，有研究^[10]^认为肺腺癌与肺鳞癌在生物学行为上的不同可能是基因表达上的差异。我们的研究发现AQP3的表达与临床病理特征存在相关，因此认为AQP3表达的明显差异可能是肺腺癌较鳞癌更容易转移的原因。AQP3的表达可能与肺腺癌关系更密切。目前，肿瘤的微血管密度被用于提示肿瘤血管生成的数量，是衡量肿瘤血管形成程度的定量指标，可以提示肿瘤的复发、转移潜能以及远期生存率，并被认为与肿瘤的生长、转移及预后有着密切的关系^[11]^。本研究发现，CD34不同程度地表达于NSCLC及癌旁正常组织，两组之间的差异明显，相关性分析表明AQP3与MVD在NSCLC组织中的表达呈正相关（*P* < 0.01），说明AQP3在NSCLC的新生血管形成过程中起着非常重要的作用，而在肿瘤的生长、转移过程中，血管的生成又为其提供了必须的物质基础和转移通道^[12]^。

本研究还发现，男性患者AQP3表达高于女性患者（*P*=0.003）。在有淋巴结转移的病例中存在AQP3高表达（*P*=0.026）。NSCLC中AQP3的阳性率与肿瘤分化程度呈明显正相关，表现为AQP3的阳性率在高分化癌中明显高于低分化癌（*P* < 0.001）。但AQP3的表达情况与年龄、病理分级等无相关性（*P* > 0.05）。既往研究^[13]^发现AQP3作为水通道中的水甘油通道参与一系列重要的物质代谢过程，在维持细胞的能量平衡中起一定作用，从而推测肿瘤细胞的能量供应、生物大分子合成等生理活动均有AQP3参与。有报道^[14]^提示，野生型小鼠与AQP3基因敲除小鼠相比，更易患肿瘤，因为AQP3缺失的小鼠的表皮细胞中甘油、3-磷酸甘油及ATP含量下降，从而使细胞增殖能力下降，减少了肿瘤发生的可能，以上研究均支持AQP3在肿瘤细胞增殖转移过程能量供应方面发挥了重要的作用。

综上所述，本研究表明，AQP3高表达与肺癌病理组织学类型、肿瘤分化程度、肿瘤性血管新生、淋巴结转移等临床特征相关。AQP3在NSCLC中的高表达可能对肿瘤血管生成起到了促进和维持的作用。因此，对AQP3的研究有助于理解肺癌组织中水电解质平衡的复杂过程，其确切机制有待于进一步研究。
